# Endocrine Resistance Score Based on Three Key Genes Predicts Prognosis and Reveals Potential Therapeutic Targets for ER+HER2− Breast Cancer

**DOI:** 10.1111/cpr.70100

**Published:** 2025-07-15

**Authors:** Liqin Ping, Lewei Zhu, Nian Chen, Xikun Liu, Jirui Zhong, Xiaoqing Sun, Hailin Tang, Kaiming Zhang

**Affiliations:** ^1^ Department of Hematology The First Affiliated Hospital of Guangzhou University of Traditional Chinese Medicine Guangzhou China; ^2^ The First People's Hospital of Foshan (The Affiliated Foshan Hospital of Southern University of Science and Technology), School of Medicine, Southern University of Science and Technology Foshan China; ^3^ The First Affiliated Hospital, Hengyang Medical School University of South China Hengyang Hunan China; ^4^ Sun Yat‐sen University Cancer Center, State Key Laboratory of Oncology in South China, Collaborative Innovation Center for Cancer Medicine, Guangdong Provincial Clinical Research Center for Cancer Guangzhou Guangdong China

**Keywords:** breast cancer, endocrine resistance, gene signature, oestrogen receptor, prognostic factors

## Abstract

Endocrine resistance is a leading cause of mortality in oestrogen receptor‐positive and human epidermal growth factor receptor 2‐negative (ER+HER2−) breast cancer (BC), highlighting the urgent need to understand its underlying molecular mechanisms and identify potentially resistant patients for effective management. In this study, we constructed endocrine‐resistant cell lines through long‐term oestrogen deprivation and identified differentially expressed genes (DEGs) via transcriptome analysis. Key endocrine‐resistant genes were defined through Cox regression analysis. Our findings revealed that the genes CLEC3A, PCDH10, and ST3GAL1 were significantly upregulated in endocrine‐resistant cells and serve as independent prognostic factors for ER+HER2− BC patients. We developed an endocrine resistance score (ERS), and a nomogram model incorporating ERS demonstrated robust predictive capabilities for patient prognosis. Single‐cell RNA sequencing analysis demonstrated that the ERS and the three core genes constituting the ERS were significantly upregulated in tissue specimens from patients with resistance to endocrine neoadjuvant therapy. Additionally, knocking down CLEC3A, PCDH10, and ST3GAL1 led to reduced malignancy progression in endocrine‐resistant BC cells. Mechanistic studies revealed that CLEC3A promotes endocrine resistance by upregulating the PI3K‐AKT pathway. This study suggests that CLEC3A, PCDH10, and ST3GAL1 are associated with endocrine resistance and can reflect the prognosis of ER+HER2− BC patients receiving endocrine therapy, providing potential therapeutic targets and a valuable prognostic indicator for clinicians.

## Graphical abstract

We generated endocrine‐resistant BC cell lines and identified CLEC3A, PCDH10, and ST3GAL1 as key endocrine‐resistant genes. More importantly, we validated their role in mediating resistance through PI3K‐AKT signalling and developed a predictive ERS with strong clinical relevance.

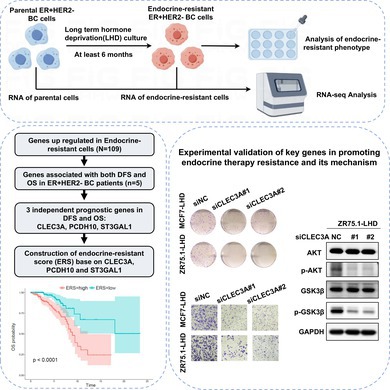

## 1 Background

Currently, the most common subtype of breast cancer (BC) is oestrogen receptor‐positive and human epidermal growth factor receptor 2‐negative (ER+HER2−) BC, accounting for 70% of the total incidence [1, 2]. The growth of ER+HER2− BC cells mainly depends on the activation of the oestrogen‐ER axis in the nucleus. Therefore, blocking this signalling axis with endocrine therapy, such as ER antagonists or aromatase inhibitors, which inhibit oestrogen synthesis, has become the effective treatment strategy for ER+HER2− BC [3]. However, up to 50% of ER+HER2− BC patients will eventually develop resistance to endocrine therapy [4, 5]. Endocrine therapy resistance is the main cause of treatment failure in ER+HER2− BC patients.

Molecular mechanisms of endocrine resistance have been partially reported. However, less than 10% of endocrine resistance is due to the loss of ER [6, 7]. Most endocrine resistance is usually driven by the ligand‐independent reactivation of ER [8]. Commonly known reasons mainly include the following four aspects: gain‐of‐function mutations in ER; compensatory crosstalk between ER and growth factor receptors and oncogenic signalling pathways; mutations and amplifications of cell cycle‐related proteins; and changes in epigenetic modifications [9, 10, 11]. In general, it is due to somatic alterations, epigenetic changes, and changes in the tumour microenvironment [11, 12, 13]. Some drugs have been approved for clinical use or have entered clinical trials, including oestrogen receptor degraders, CDK4/6 inhibitors, histone deacetylase (HDAC) inhibitors, and PI3K/AKT/mTOR pathway inhibitors [11, 14, 15]. However, single‐agent therapies such as Elacestrant demonstrate a median progression‐free survival (PFS) of merely 2.8 months in patients with endocrine‐resistant BC [16]. Similarly, Abemaciclib, a CDK4/6 inhibitor, shows limited activity as a monotherapy with a median PFS of 6 months [17]. Combination strategies, including Tucidinostat plus exemestane (median PFS 7.4 months [18]) and Capivasertib (a PI3K/p‐AKT inhibitor) plus fulvestrant (median PFS 7.2 months [19]), also exhibit modest improvements. These data highlight the ongoing challenges in overcoming endocrine resistance and underscore the need for further advancements in therapeutic strategies for this patient population [20]. Additionally, it is reported that 60% of endocrine‐resistant BC lack known resistance‐driving factors, and these patients do not have suitable targeted drugs available [21]. Moreover, ER+HER2− BC exhibits significant heterogeneity, with large variations in response to endocrine therapy. Therefore, identifying underlying molecular mechanisms of endocrine resistance, developing biomarkers based on resistance mechanisms, and molecularly stratifying patients will be of guiding significance for the clinical management of these patients. It will also provide references for exploring new approaches to endocrine resistance treatment.

In this study, we constructed endocrine‐resistant BC cell lines by long‐term oestrogen deprivation culture. Then, we identified some key genes that played a part in endocrine resistanceto predict the prognosis of patients with ER+HER2− BC. In addition, we explored the role of these key genes in tumour malignancy progression through in vitro experiments.

## 2 Methods

### 2.1 Establishment of Endocrine Therapy‐Resistant Cell Lines

The human ER+HER2− BC cell lines (MCF7 and ZR75.1) were obtained from the Cell Bank of the Chinese Academy of Sciences (Shanghai, China). The parental MCF7 [22] and ZR75.1 cells were cultured in DMEM medium containing 10% fetal bovine serum (FBS) at 37°C in a 5% CO_2_ cell culture incubator. Aromatase inhibitors (AI) are the primary endocrine therapy agents used in BC treatment. Their mechanism of action involves the inhibition of oestrogen synthesis, thereby reducing systemic oestrogen levels and suppressing tumour growth through oestrogen receptor signalling. To mimic the therapeutic effects of AI, we established an in vitro oestrogen‐deprived model to evaluate the impact of exogenous oestrogen withdrawal on BC cell behaviour under experimental conditions. Oestrogen is removed from the FBS using activated carbon [23, 24]. Then, the MCF7 and ZR75.1 cells were cultured in oestrogen‐deprived medium for at least 6 months until they could proliferate rapidly in the oestrogen‐deprived medium [25]. The resulting endocrine‐resistant cell lines were named MCF7‐long‐term oestrogen deprivation (LHD) and ZR75.1‐LHD.

### 2.2 Differentially Expressed Genes (DEGs)

Transcriptome sequencing was performed on the parental cell lines (MCF7 and ZR75.1) and the endocrine‐resistant cell lines (MCF7‐LHD and ZR75.1‐LHD). Analysis of differentially expressed genes (DEGs) was conducted using the “DEseq2” R package, with genes showing a |log2FC| > 1 and *p* < 0.05 considered as differentially expressed. Subsequently, Gene Ontology (GO) enrichment analysis was performed using the “Clusterprofiler” package with an adjusted *p* value < 0.05 set as the threshold [26].

### 2.3 Endocrine Resistance‐Related Biomarker

Volcano plots were generated using ggplot2 to visualise the DEGs between MCF7 and MCF7‐LHD, as well as ZR75.1 and ZR75.1‐LHD. The core genes of endocrine resistance are defined as those that are co‐upregulated in MCF7‐LHD and ZR75.1‐LHD. A Venn diagram was used to analyse commonly upregulated genes between the two pairs of cell lines. The TCGA (https://cancergenome.nih.gov/) database is employed to analyse the impact of key genes on the survival of ER+HER2− BC patients. Cox regression analysis was conducted to identify independent prognostic endocrine‐resistant genes. Endocrine Resistance Score (ERS) was constructed based on the proportional hazards' regression analysis of endocrine‐resistant genes in the TCGA database.

### 2.4 Prognostic Prediction Ability of ERS for ER+HER2− BC Patients

To validate the prognostic prediction ability of ERS for ER+HER2− BC patients, the METABRIC database (http://molonc.bccrc.ca/aparicio‐lab/research/metabric/) is employed as an external validation cohort. Patients were stratified into ERS‐high and ERS‐low groups based on the median value. Survival was analysed using the Kaplan–Meier method. Between‐group comparisons were conducted using the log‐rank test. The prognostic value of ERS, along with other clinical features (such as age, T stage, and N stage) was explored through univariate and multivariate Cox regression analysis.

### 2.5 Analysis of FELINE scRNAseq

The single‐cell sequencing analysis methods were described in a prior study [27]. The 10X single‐cell RNA sequencing (scRNA‐seq) count data from the FELINE study were downloaded from GEO (accession GSE158724). Eight post‐neoadjuvant letrozole‐treated surgical samples were analysed, including four samples from responders and four from non‐responders. The expression levels of CLEC3A, ST3GAL1, and PCDH10 were compared in tumour cells from responders and non‐responders. The ERS score was calculated based on the expression levels of these three genes, and the ERS scores of responders and non‐responders were compared.

### 2.6 Gene Set Enrichment Analysis and Immune Infiltration

DEGs between ERS‐high and ERS‐low groups were identified using the “DEseq2” R package. GSEA was employed to investigate signalling pathways between ERS‐high and ERS‐low patients. The CIBERSORT algorithm was utilised to analyse the proportions of tumour‐infiltrating immune cells. Differences in immune cells between ERS‐high and ERS‐low groups were compared. Results from the CIBERSORT algorithm were filtered based on a significance level of *p* < 0.05.

### 2.7 mRNA Expression Levels

Total RNA was extracted using RNA Purification Kit (B0004D, EZBioscience), and reverse transcription was performed using a HiScript II Q RT Kit (R223‐01, Vazyme Biotech). Real‐time PCR was performed using ChamQ SYBR qPCR Green Master Mix (Q311‐03, Vazyme Biotech) and run with a Light Cycler 480 instrument (Roche Diagnostics) [28]. All qPCR reactions were performed in triplicate, and the relative expression of the target gene mRNA was normalised to GAPDH. Primers for CLEC3A, PCDH10, ST3GAL1, and GAPDH were as follows:

- Human CLEC3A sense: 5′‐TCACCTTACTCCTGGACCAGAC‐3′,
- Human CLEC3A antisense: 5′‐GGCATTGACTTCTGTCCAGAGC‐3′;
- Human PCDH10 sense: 5′‐TCTCCAACGGAAGCATTTTGTCC‐3′,
- Human PCDH10 antisense: 5′‐CTATGTCGGCTTCCTGGAATGC‐3′;
- Human ST3GAL1 sense: 5′‐TTGGAGGACGACACCTACCGAT‐3′,
- Human ST3GAL1 antisense: 5′‐CACCACTCTGAACAGCTCCTTG‐3′;
- Human GAPDH sense: 5′‐GATTCCACCCATGGCAAATTC‐3′,
- Human GAPDH antisense: 5′‐CTTCTCCATGGTGGTGAAGAC‐3′.

### 2.8 Gene Knockdown

Cells were seeded in 6‐well plates and siRNA transfection was performed when MCF7‐LHD and ZR75.1‐LHD reached 30%–50% confluence. Transfection of siRNA was performed with lipofectamine RNAimax (Invitrogen) according to the manufacturer's instructions. The siRNA target sequences for CLEC3A, ST3GAL1, or PCDH10 were as follows:

- CLEC3A#1(human): 5′‐GCACTAAAGTTCACAAGAAAT‐3′,
- CLEC3A#2(human): 5′GCCCTCCAAGACTATGGTAAA‐3′,
- ST3GAL1#1(human): 5′‐GATGCAGACTTTGAGTCTAAC‐3′,
- ST3GAL1#2(human): 5′‐GCTGGGAGATAATGTCAGCAT‐3′,
- PCDH10#1(human): 5′‐TAAGCGGCGAGTTGGACTATG‐3′,
- PCDH10#2(human): 5′‐GCCACTGTCAACATCCTCATA‐3′.

### 2.9 BrdU Incorporation Assay

Cells were seeded in 12‐well plates (5 × 10^5^ cells/well). Twenty‐four hour post‐seeding, the medium was replaced with BrdU‐contained medium at a final concentration of 10 μM for 4 h. Then, cells were fixed with 70% ethanol for 30 min at 4°C. Fixed cells were resuspended in 0.1% Triton X‐100 in PBS to permeabilise the cell membrane, followed by incubation at 80°C for 20 min to denature DNA and expose BrdU. After that, cells were incubated with anti‐BrdU antibody (PE Conjugate) anti‐BrdU primary (1:100 dilution, Cell Signalling Technology, #50230) for 1 h at 4°C. After washing, cells were resuspended in 0.5 mL PBS and analysed on a flow cytometer.

### 2.10 Colony Formation Assay

Cells were seeded at a low density (500 cells/well) in 6‐well plates and treated or manipulated according to the experimental design. Following treatment, cells were cultured for 10 days. After the incubation period, colonies were fixed with 4% paraformaldehyde fix solution for 30 min and stained with crystal violet (0.5% in methanol) for 20 min. Then, unbound dye was rinsed off with phosphate‐buffered saline (PBS) under gentle continuous flow. The plates were air‐dried completely at room temperature, after which colony formation was imaged using a digital camera system with uniform lighting and focus to ensure clear colony boundaries. The colony formation assay images were analysed using ImageJ (version 1.53t; NIH, USA) to quantify colony formation as an indicator of cell survival and proliferation.

### 2.11 MTT Assay and Transwell Assay

The 3‐(4,5‐dimethyl‐2‐thiazolyl)‐2,5‐diphenyl‐2H‐tetrazolium bromide (MTT) assay was utilised for assessing cell viability [29, 30]. Parental and endocrine‐resistant cell lines were seeded in 96‐well plates. At baseline and following culture for 2, 4, 6, and 8 days, the cells were co‐incubated with 1 mg/mL MTT solution for 4–6 h. Subsequently, absorbance at a wavelength of 450 nm was measured. The Transwell assay is employed to evaluate the migratory capacity of cells. The Transwell device was placed in a culture dish, with cells added to the upper chamber and medium in the lower chamber. The Transwell device was then placed in a culture incubator to allow the cells to adhere and migrate or invade [31, 32]. After a period of time, the presence of cell migration or invasion in the lower chamber was observed, which could be assessed through staining. Samples were collected for staining and cell counting to compare the migration capability.

### 2.12 Immunoblot

The process of immunoblot and immunoprecipitation was described previously [33, 34]. Briefly, cells were harvested and lysed in RIPA buffer (Cat#9806s, Cell Signaling Technology) containing 1 mM phenylmethanesulfonyl fluoride. Total proteins were separated by SDS‐PAGE and transferred to PVDF membrane. Antibodies for immunoblot were used at a dilution of 1:500–1:1000.

### 2.13 Immunohistochemical Staining

The process of immunohistochemical staining was described previously [35, 36]. Briefly, patients who experienced tumour recurrence or progression within 3 years of adjuvant endocrine therapy were defined as endocrine therapy‐resistant. In contrast, patients who remained recurrence‐free with no evidence of tumour progression for 5–10 years during adjuvant endocrine therapy were classified as endocrine therapy‐sensitive. Thirty‐nine paraffin blocks of human ER+HER2− breast lesions were selected for this study. These samples were histopathologically and clinically diagnosed as ER+HER2− BC at the Sun Yat‐sen University Cancer Center. Seventeen patients were classified as endocrine therapy‐resistant and 22 patients were classified as endocrine therapy‐sensitive. All samples used in this study were approved by the medical ethics committee of Sun Yat‐sen University Cancer Center. For evaluation of CLEC3A/PCDH10/ST3GAL1 staining, we adopted a staining index by multiplying the score for the percentage of positive tumour cells by the intensity score, which was obtained as the intensity staining (0, no staining; 1, weak; 2, moderate; 3, strong) and the percentage of positive cells (1, 0%–25%; 2, 26%–50%; 3, 51%–75%; 4, 76–%100%). Sections with a final score < 3 were considered low CLEC3A expression, whereas sections with a final score ≥ 3 were considered high CLEC3A expression. Sections with a final score < 3 were considered low PCDH10 expression, whereas sections with a final score ≥ 3 were considered high PCDH10 expression. Sections with a final score < 4 were considered low ST3GAL1 expression, whereas sections with a final score ≥ 4 were considered high ST3GAL1 expression. A chi‐squared test was performed to assess statistically significant differences in immunohistochemical staining scores between endocrine therapy‐sensitive and ‐resistant patients.

### 2.14 Statistical Analysis

R software (version 4.0.3) and GraphPad Prism 6.0.1 (GraphPad, La Jolla, CA, USA) were used to conduct the statistical analyses. Survival curves were plotted by the Kaplan–Meier method in R software. The *p* values were assessed using the log‐rank test. Cox regression analysis was used to determine independent prognostic factors. The results presented as the mean ± SD were analysed by one‐way ANOVA with Tukey's multiple comparisons test using GraphPad Prism. All the statistical tests were two‐sided; *p* < 0.05 was considered statistically significant.

## 3 Results

### 3.1 Construction of Endocrine‐Resistant Cell Lines

**FIGURE 1:**
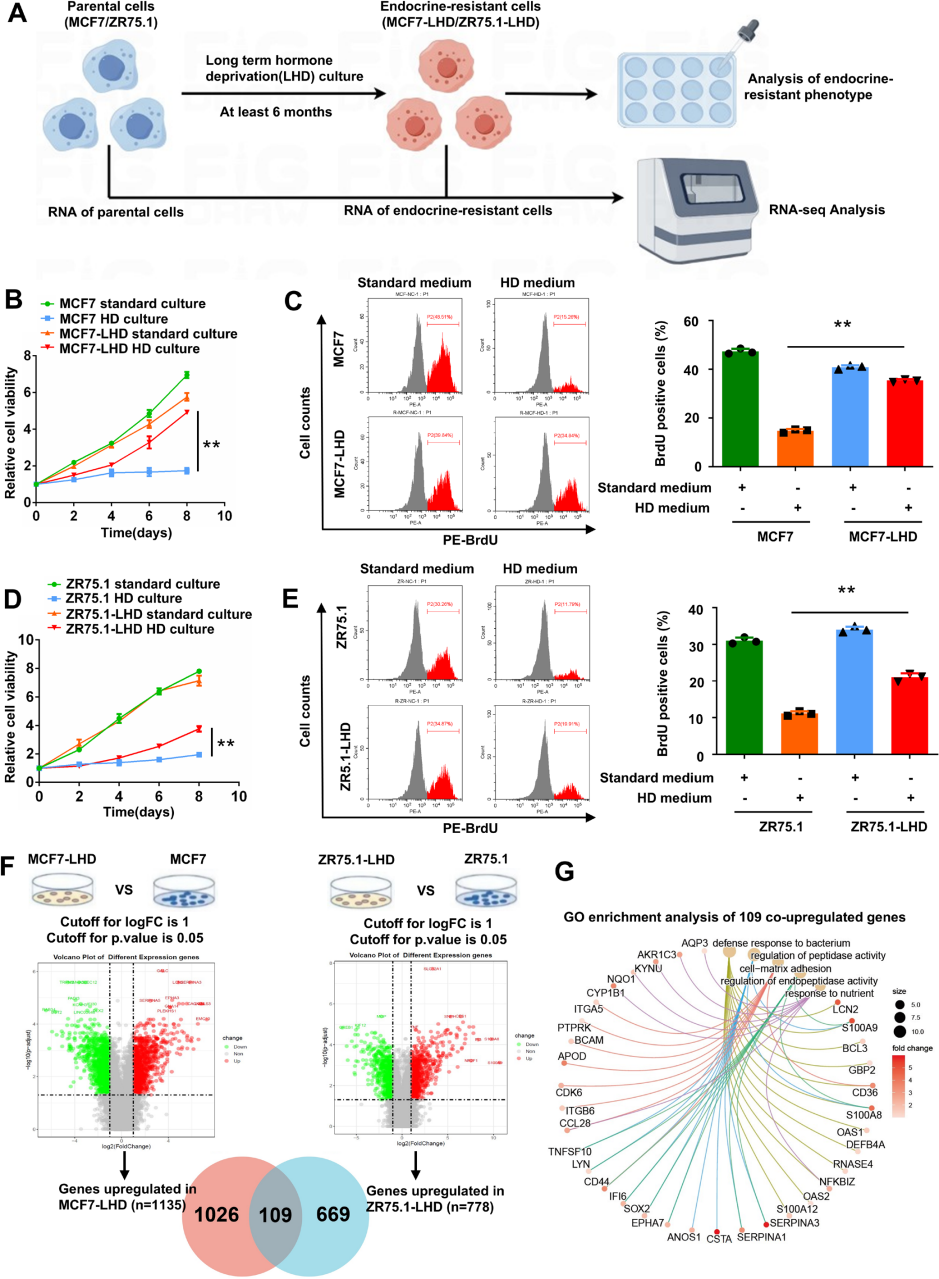
Construction and validation of endocrine‐resistant breast cancer cell Lines (MCF7‐LHD/ZR75.1‐LHD). (A) The MCF7 and ZR75.1 cell lines were cultured in oestrogen‐deprived medium for at least 6 months to establish endocrine‐resistant cell lines. (B, C) Cell viability curves and BrdU cell proliferation assay of MCF7 and MCF7‐LHD cells in standard and oestrogen‐deprived culture media. (D, E) Cell viability curves and BrdU cell proliferation assay of ZR75.1 and ZR75.1‐LHD cells in standard and oestrogen‐deprived culture media. (F) Identify the differentially expressed genes that are commonly upregulated in MCF7‐LHD and ZR75.1‐LHD. (G) Gene Ontology (GO) enrichment analysis of the upregulated differentially expressed genes in the endocrine‐resistant cell lines. The cell viability curves and colony formation assays were repeated in three independent experiments. Error bars represent the standard error of the mean (**p < 0.01).

To identify the biological characteristics and underlying mechanisms of endocrine resistance, we established endocrine‐resistant cell models through long‐term hormone deprivation culture (Figure 1A). The MTT cell proliferation assay and BrdU cell proliferation assay showed that the cell proliferation of the endocrine‐resistant cell MCF7‐LHD was significantly faster than that of the parental cell MCF7 in the hormone deprivation medium (Figure 1B,C). Additionally, the colony formation ability of parental cell MCF7 was significantly decreased in the hormone deprivation medium, while the colony formation ability of MCF7‐LHD was not significantly inhibited by the deprivation of hormone (Figure S1A). Similarly, the proliferation and colony formation ability of ZR75.1‐LHD in hormone deprivation medium were significantly higher than those of the parent cell line ZR75.1 (Figures 1D,E and S1B).

### 3.2 Transcriptome Analysis of Parental and Endocrine‐Resistant BC Cells

Subsequently, we conducted transcriptome sequencing on both the parental cells (MCF7 and ZR75.1) and endocrine‐resistant cells (MCF7‐LHD and ZR75.1‐LHD). The results of the DEGs analysis revealed that compared with parental cells, 1135 genes were upregulated in the MCF7‐LHD cell and 778 genes were upregulated in the ZR75.1‐LHD cell. Furthermore, we pinpointed 109 genes that were co‐upregulated in both the MCF7‐LHD and ZR75.1‐LHD cells (Figure 1F and Table S1). Then, we subjected these 109 co‐upregulated DEGs to GO enrichment analysis. The results of the enrichment analysis indicated that the upregulation of pathways in endocrine‐resistant BC cells was related to defence response to bacterium, cell‐matrix adhesion, and regulation of endopeptidase activity (Figure 1G).

### 3.3 Identification of Co‐Upregulated DEGs Associated With Survival

**FIGURE 2:**
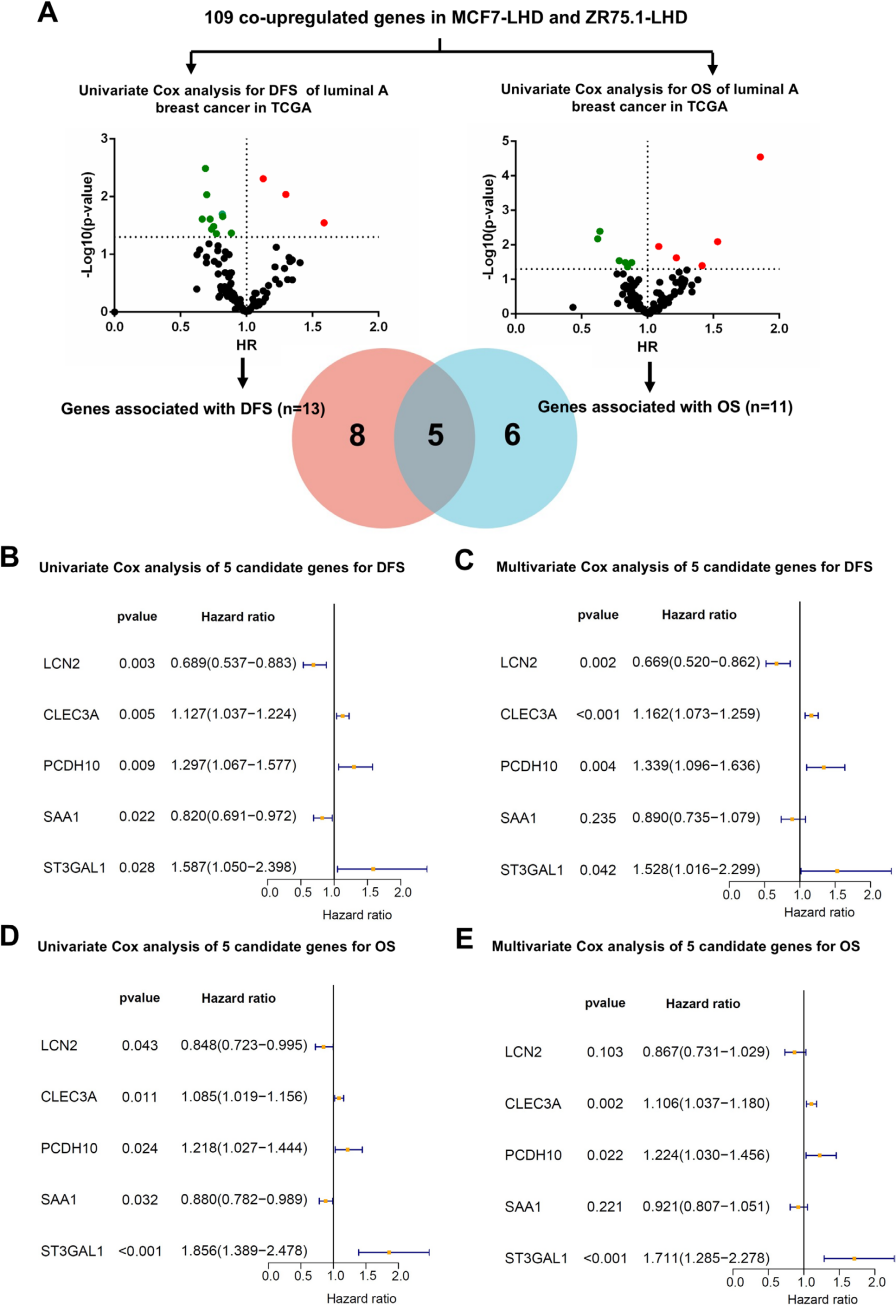
Identification of endocrine resistance‐associated Genes. (A) Identification of genes that are commonly upregulated in MCF7‐LHD and ZR75.1‐LHD cells, and that impact survival (DFS and OS) in TCGA database. (B, C) Univariate and multivariate Cox regression analysis of candidate genes for DFS. (D, E) Univariate and multivariate Cox regression analysis of candidate genes for OS.

To identify key genes in endocrine‐resistant BC, we analysed the prognostic value of co‐upregulated DEGs in patients with luminal A BC. The results showed that among the 109 co‐upregulated genes, 13 genes impacted patients' disease‐free survival (DFS), while 11 genes influenced overall survival (OS) (Figure 2A). By identifying the intersection of genes affecting both DFS and OS, we pinpointed five endocrine‐resistant genes (Figure 2A and Table S2). To further ascertain the impact of these genes on patients' survival, we conducted univariate and multivariate Cox regression analyses on these five genes. We found that the genes LCN2, CLEC3A, PCDH10, and ST3GAL1 were independent prognostic factors for DFS of BC patients (Figure 2B,C). Additionally, CLEC3A, PCDH10, and ST3GAL1 genes emerged as independent prognostic factors for OS (Figure 2D,E).

### 3.4 Construction and Validation of ERS

**FIGURE 3:**
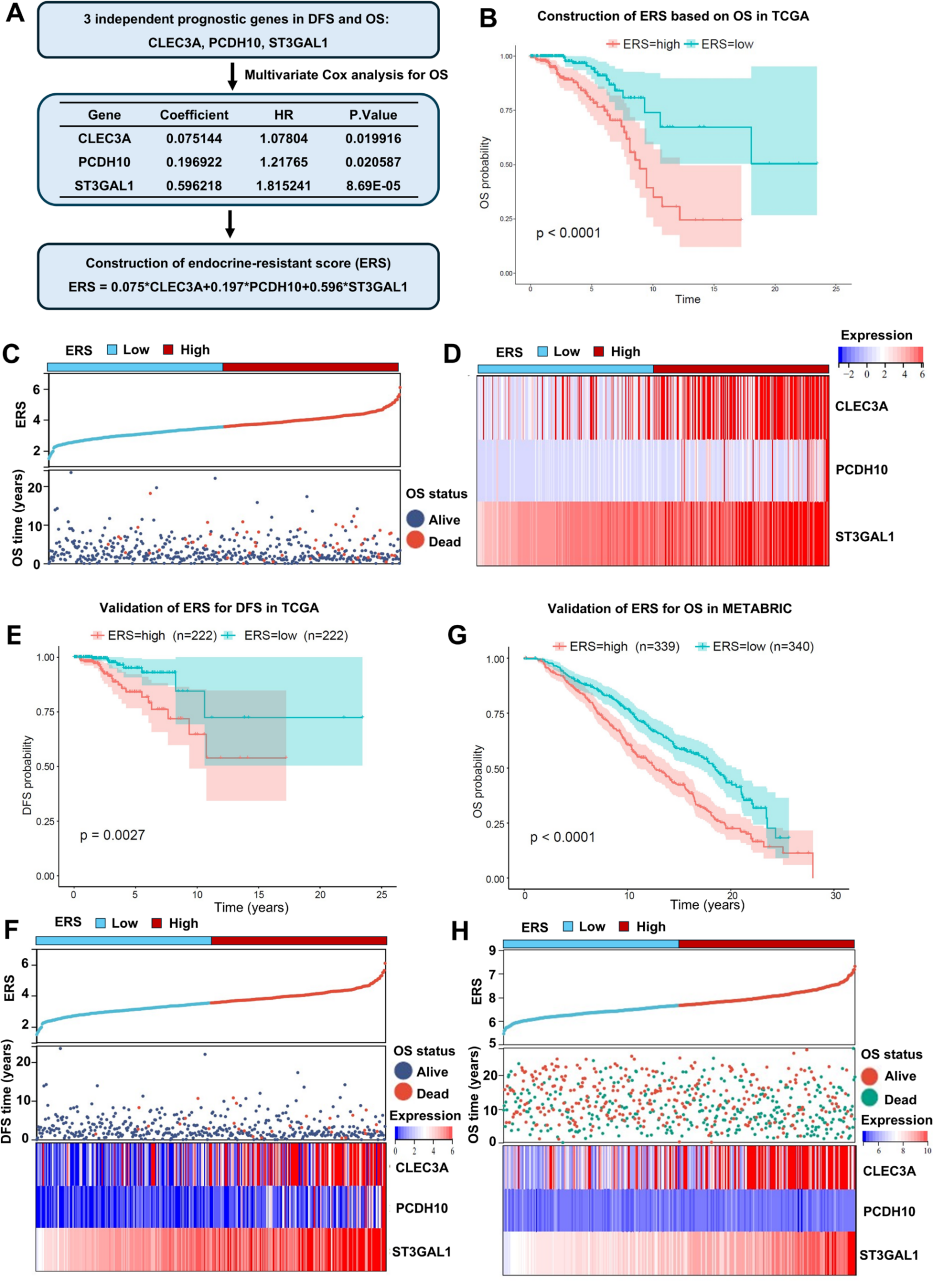
Construction of endocrine resistance score. (A) Multivariate Cox regression analysis was performed on the three identified endocrine resistance‐related genes to calculate their coefficient values, which were then used to calculate the Endocrine Resistance Score (ERS). (B, C) Comparison of OS and survival status between patients with high and low ERS in the TCGA database. (D) Expression levels of key endocrine resistance genes CLEC3A, PCDH10, and ST3GAL1 in patients with high and low ERS. (E, F) Comparison of DFS, DFS status, and expression of key endocrine resistance genes in patients with high and low ERS in the TCGA database (*n* = 510). (G, H) Comparison of OS, OS status, and expression of key endocrine resistance genes in patients with high and low ERS in the MRTABRIC database (*n* = 679).

We then employed key endocrine‐resistant genes to construct a predictive model to predict the response to endocrine therapy in ER+HER2− BC patients and ultimately guide precision therapy. The genes CLEC3A, PCDH10, and ST3GAL1 were identified as independent prognostic factors for both DFS and OS in ER+HER2− BC patients. So, these three genes were subjected to multivariate regression analysis to establish an ERS formula: ERS = 0.075 * expression of CLEC3A + 0.197 * expression of PCDH10 + 0.596 * expression of ST3GAL1 (Figure 3A). Patients were stratified into ERS‐high and ERS‐low groups based on the median ERS value in the TCGA database. Patients in the ERS‐high group exhibited significantly worse OS than those in the ERS‐low group (Figure 3B). The distribution of ERS, survival time, and survival status of OS in the TCGA database are shown in Figure 3C. Expression levels of CLEC3A, PCDH10, and ST3GAL1 were significantly higher in the ERS‐high group compared to the ERS‐low group (Figure 3D). Then, longer DFS was observed in the ERS low group of ER+HER2− BC patients in the TCGA database (Figure 3E,F). In addition, the prediction ability of ERS was also externally validated by the OS of ER+HER2− BC patients in the METABRIC database. Consistently, the ERS low group of ER+HER2− BC patients in the METABRIC database had significantly longer OS than ERS‐high group (Figure 3G,H).

### 3.5 ERS Is a Biomarker for Endocrine Resistance and Independent Prognostic Factor

**FIGURE 4:**
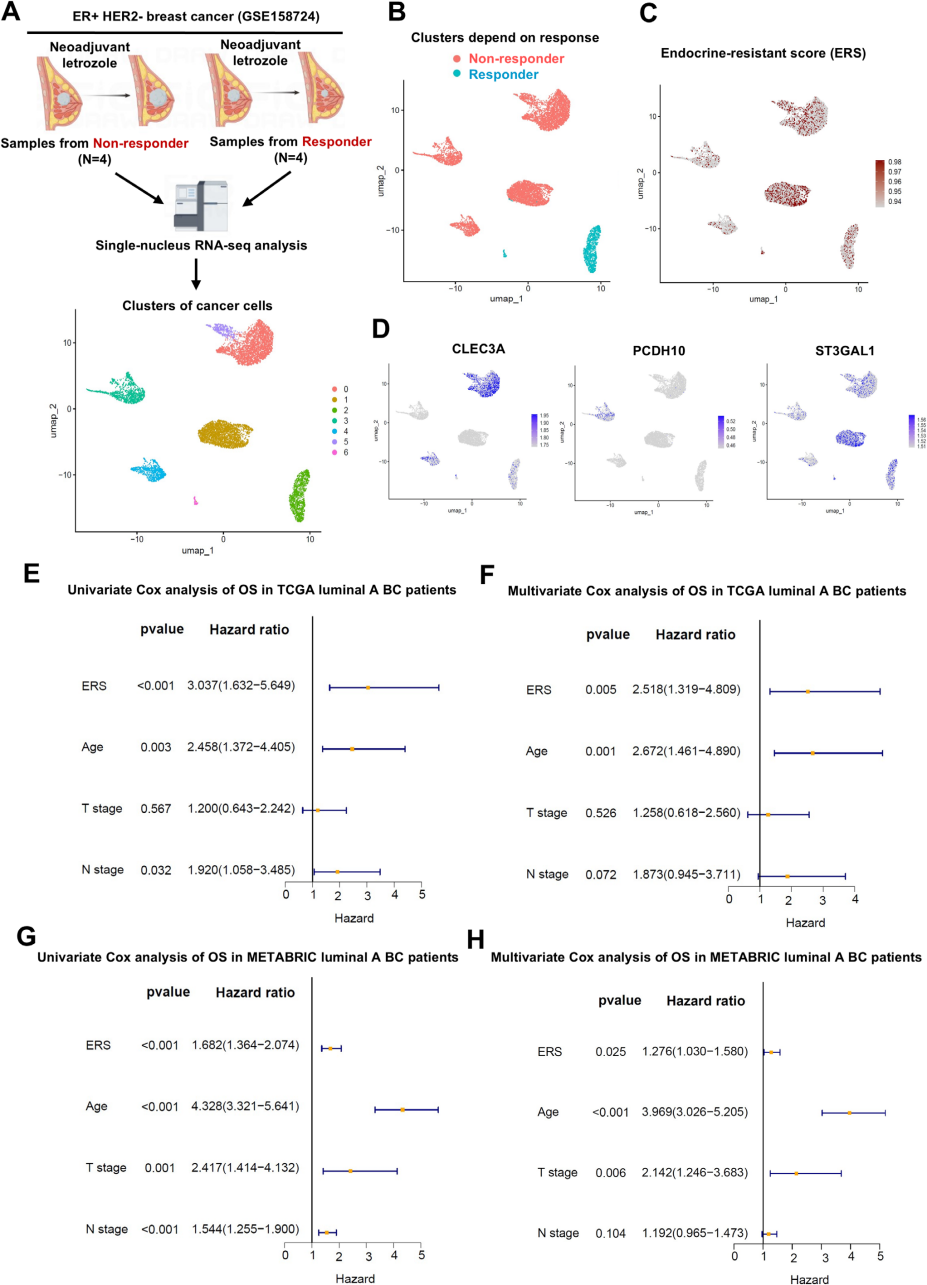
Prognostic significance of endocrine resistance score in luminal breast cancer. (A) Single‐cell RNA sequencing analysis of eight post‐neoadjuvant letrozole‐treated samples. (B) Tumour cell clusters from responders and non‐responders. (C) ERS was higher in tumour cells from non‐responders. (D) Higher expression of CLEC3A, ST3GAL1, and PCDH10 was observed in non‐responders. (E, F) Univariate and multivariate Cox regression analysis of ERS and clinical parameters for OS in luminal breast cancer patients in the TCGA database. (G, H) Univariate and multivariate Cox regression analysis of ERS and clinical parameters for OS in luminal breast cancer patients in the MRTABRIC database.

**FIGURE 5:**
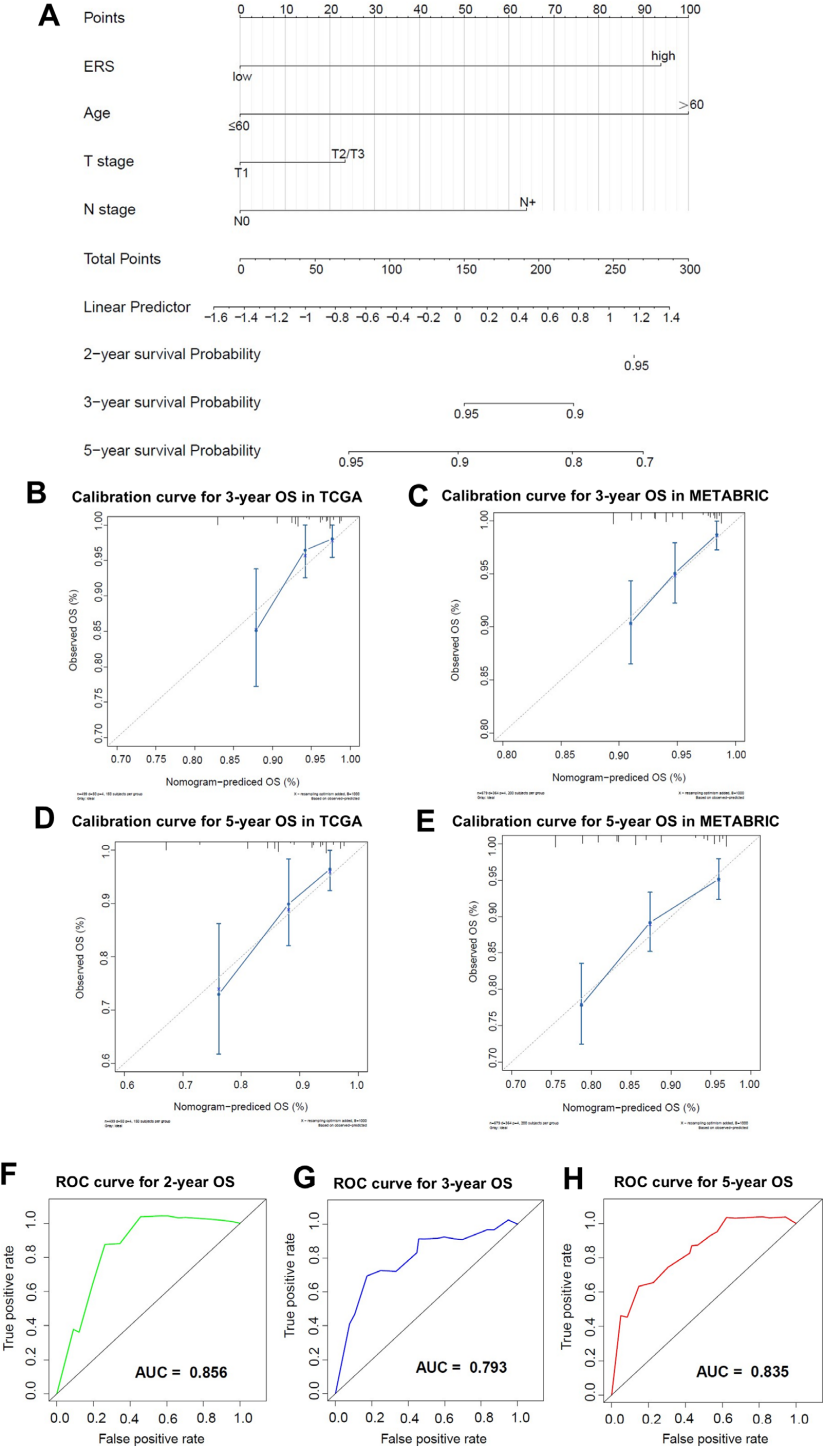
Construction of a nomogram prognostic model including ERS. (A) A nomogram prognostic prediction model incorporating ERS, age, T stage, and N stage was constructed using TCGA database. (B, C) Calibration plots for 3‐year OS in TCGA and METABRIC databases. (D, E) Calibration plots for 5‐year OS in TCGA and METABRIC databases. (F–H) Validation of the nomogram model for 2‐year, 3‐year, and 5‐year OS in the TCGA database.

To further investigate the relationship between ERS and resistance to endocrine therapy, single‐cell RNA sequencing data of eight post‐neoadjuvant letrozole‐treated surgical samples from the FELINE study were analysed(Figure 4A). The tumour cell clusters derived from responders and non‐responders were clearly separated (Figure 4B), and the ERS calculated for each cell showed that the tumour cells derived from non‐responders had a significantly higher ERS than those derived from responders (Figure 4C). Further analysis of the expression levels of three key genes in the ERS (CLEC3A, ST3GAL1, and PCDH10) revealed that these genes were significantly more highly expressed in tumour cells from non‐responders than in those from responders (Figure 4D). Subsequently, we investigated the prognostic predictive value of ERS in OS of ER+HER2− BC patients. ERS, age, T stage, and N stage were included in a Cox regression analysis. The results demonstrated that in the TCGA database, higher ERS scores were independently associated with poorer OS of ER+HER2− BC patients (Figure 4E,F). Consistently, ERS was also an independent prognostic factor for OS of ER+HER2− BC patients in the METABRIC database (Figure 4G,H). To further utilise ERS to precisely predict endocrine resistant BC patients, we constructed a nomogram model that included ERS, age, T stage, and N stage (Figure 5A). The calibration curve showed high consistency at 3 and 5 years (Figure 5B–E), suggesting that the predictive ability of the nomogram based on ERS is reliable. In addition, the results of the time‐dependent receiver operating characteristic (ROC) curve revealed that the nomogram based on ERS had an area under the curve (AUC) of 0.856, 0.793, and 0.835 for predicting 2‐year, 3‐year, and 5‐year OS of ER+HER2− BC patients, respectively (Figure 5F–H).

### 3.6 Gene Set Enrichment Analysis and Immune Infiltration Cells

**FIGURE 6:**
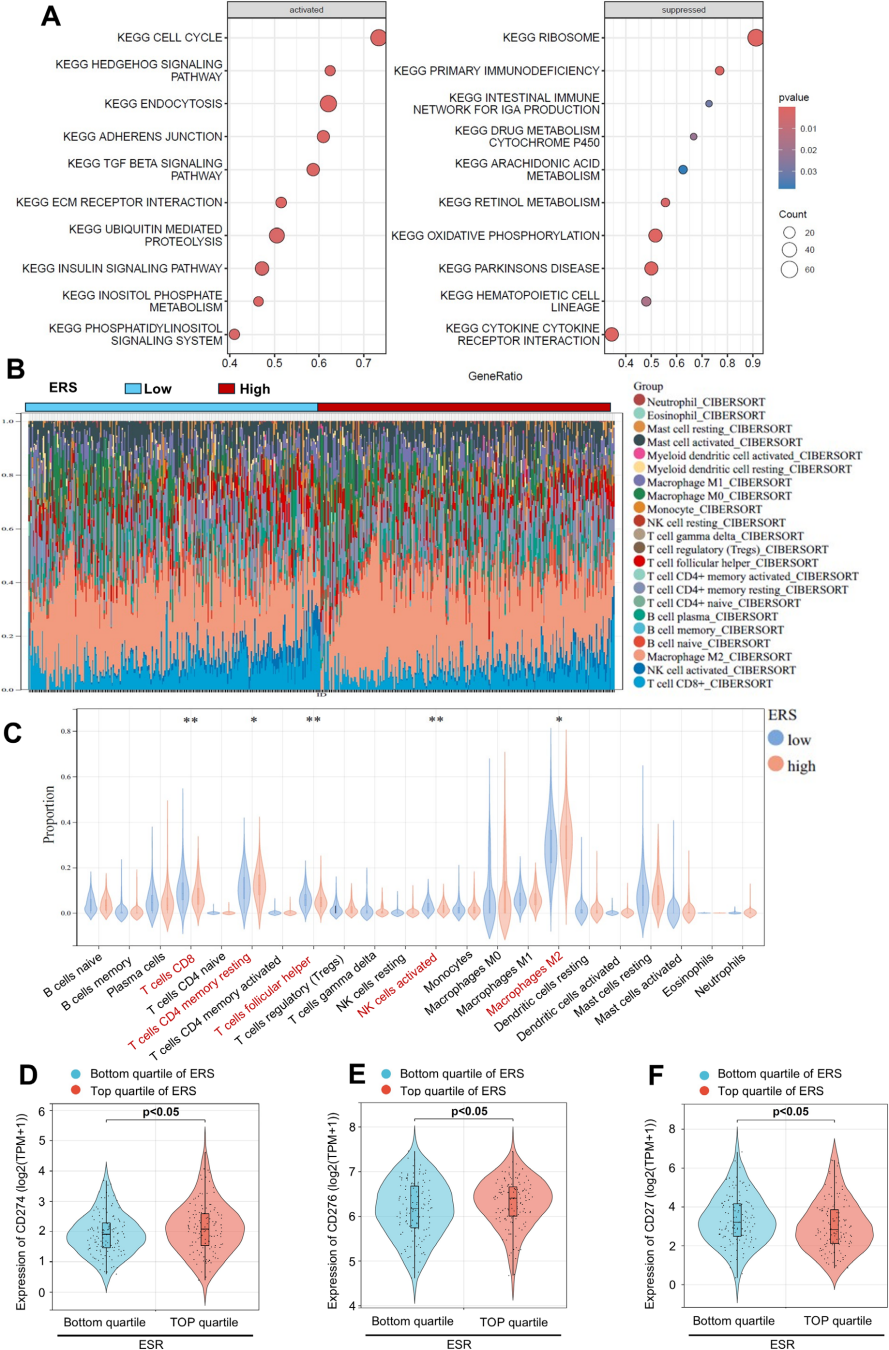
Endocrine resistance‐related signalling pathways and microenvironment. (A) Activated and suppressed cell signalling pathways in ERS‐high versus ERS‐low patients. (B) Immune cell infiltration in the immune microenvironment of ERS‐high versus ERS‐low patients. (C) Violin plots comparing immune cell infiltration in the immune microenvironment of ERS‐high and ERS‐low patients. (D–F) Violin plots comparing the expression of CD274, CD246 or CD27 in top quartile ERS group and bottom quartile ERS group.

To further explore the signalling pathways associated with ERS, we conducted Gene Set Enrichment Analysis (GSEA) between patients in ERS‐high and ERS‐low groups. The results revealed that upregulated pathways in ERS‐high patients were cell cycle, hedgehog signalling pathway, endocytosis, and adherens junction (Figure 6A). Conversely, ribosome, primary immunodeficiency, and intestinal immune network for IgA production were downregulated in ERS‐high patients. Additionally, Cibersort analysis indicated significant differences in immune‐infiltrating cells between ERS‐high and ERS‐low BC patients (Figure 6B). Patients in the ERS‐low group exhibited higher infiltration of CD8+ T cells and activated NK cells, while M2‐type macrophages were lower (Figure 6C). Further analysis revealed that patients with the top quartile of ERS exhibited significantly higher expression of the immune checkpoints CD274 and CD276 compared to those in the bottom quartile of ERS (Figure 6D,E). Conversely, patients in the top quartile group showed significantly lower expression of the immune co‐stimulatory molecule CD27 compared to the bottom quartile group (Figure 6F). Taken together, these results suggested that ER+HER2− BC with high ERS tended to be characterised by accelerated cell cycle progression and the presence of immunosuppressive tumour microenvironments.

### 3.7 Silencing Key Endocrine Resistance Genes Inhibits Tumour Malignant Progression

**FIGURE 7:**
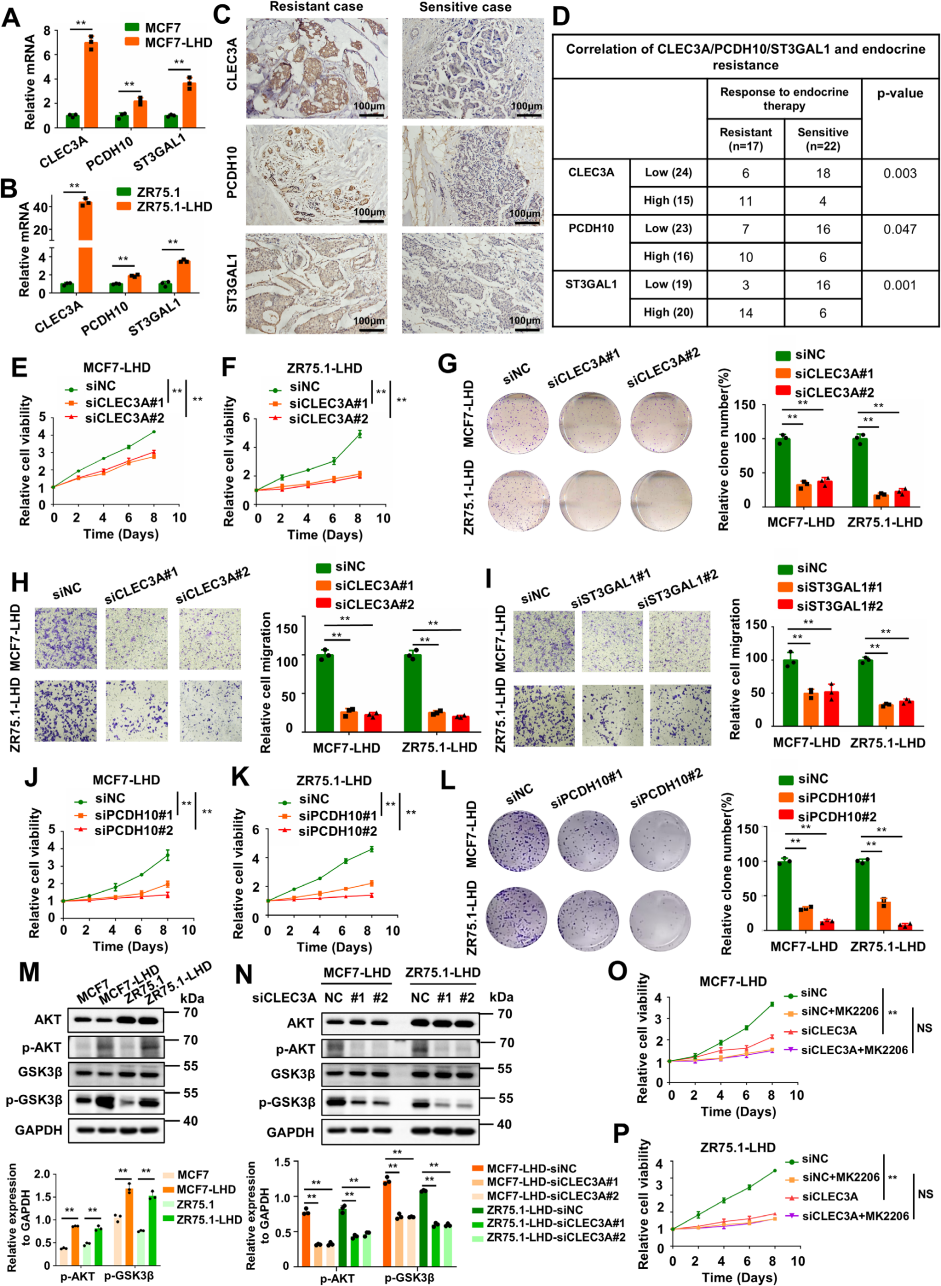
In vitro exploration of the functional roles of key genes associated with endocrine resistance. (A, B) Comparison of CLEC3A, PCDH10, and ST3GAL1 expression in parental versus endocrine‐resistant cell lines. (C) Representative IHC of CLEC3A, PCDH10, and ST3GAL1 for patients with endocrine‐resistant or sensitive breast cancer. Scale bar, 100 μm. (D) Correlations of CLEC3A, PCDH10, and ST3GAL1 expression levels with response to endocrine therapy (*χ* ^2^ test; *p* values are indicated). (E, F) Silencing CLEC3A in MCF7‐LHD and ZR75.1‐LHD cell lines results in a substantial impairment of cell proliferative potential. (G, H) Reduced colony formation and invasion ability following CLEC3A knockdown in MCF7‐LHD and ZR75.1‐LHD cell lines. (I) Knockdown of ST3GAL1 in MCF7‐LHD and ZR75.1‐LHD cell lines leads to a reduction in invasive ability. (J, K) Silencing PCDH10 in MCF7‐LHD and ZR75.1‐LHD cell lines results in a substantial impairment of cell proliferative potential. (L) Reduced colony formation ability following PCDH10 knockdown in MCF7‐LHD and ZR75.1‐LHD cell lines. (M) MCF7/ZR75.1 cells and R‐MCF7/R‐ZR75.1 cells were harvested for western blot analysis of proteins in PI3K‐AKT pathway. (N) Knockdown of CLEC3A in MCF7‐LHD and ZR75.1‐LHD cell lines leads to a suppression of PI3K‐AKT pathway. (O, P) MTT cell proliferation assay for MCF7‐LHD and ZR75.1‐LHD treated with siCLEC3A and/or MK2206. Error bars represent the standard error of the mean (**p < 0.01).

Further analysis showed that patients with high ERS exhibited significantly higher expression levels of CLEC3A, PCDH10, and ST3GAL1 genes (Figure S2A). Through RT‐qPCR, it was found that the expression of these three genes was significantly upregulated in the MCF‐LHD and ZR75.1‐LHD cell lines (Figure 7A,B). To investigate the clinical implications of CLEC3A, PCDH10, and ST3GAL1 expression in ER+HER2− BC patients with endocrine resistance, we analysed their expression profiles in 39 BC tissues via immunohistochemistry (IHC) (Figure 7C). Our findings revealed significantly elevated expression of CLEC3A (*p* = 0.003), PCDH10 (*p* = 0.047), and ST3GAL1 (*p* = 0.001) in endocrine‐resistant tumour samples compared to endocrine‐sensitive controls (Figure 7D). Then, we employed siRNA to knock down CLEC3A, PCDH10, and ST3GAL1 genes in MCF7‐LHD and ZR75.1‐LHD cells (Figure S2B–D). The results of the cell proliferation assay showed that CLEC3A knockdown significantly inhibited the proliferation rate of MCF7‐LHD/ZR75.1‐LHD in hormone‐deprived medium (Figure 7E,F). In addition, CLEC3A knockdown reduced the colony formation ability of MCF7‐LHD and ZR75.1‐LHD (Figure 7G). Transwell migration assays were performed to examine the migration capability of endocrine‐resistant BC cells, and we found that CLEC3A knockdown significantly suppressed the migration ability of MCF7‐LHD and ZR75.1‐LHD cells (Figure 7H). Unlike CLEC3A, ST3GAL1 knockdown did not affect the proliferation rate or colony formation ability of MCF7‐LHD and ZR75.1‐LHD (Figure S2E–G), but ST3GAL1 knockdown significantly inhibited the migration ability of MCF7‐LHD and ZR75.1‐LHD cells (Figure 7I). Consistent with CLEC3A, knockdown of PCDH10 significantly inhibited proliferation and colony formation potential in endocrine‐resistant BC cell lines MCF7‐LHD and ZR75.1‐LHD (Figure 7J,K,L). Functional experiments demonstrated that CLEC3A enhanced colony formation and migratory capacity in endocrine‐resistant BC cells, suggesting its critical role in promoting endocrine therapy resistance. Motivated by these findings, we sought to delineate the underlying molecular mechanism. Based on previous studies showing that the PI3K‐AKT pathway is a dominant driver of endocrine therapy resistance in clinical BC samples and preclinical models [37], we hypothesized a functional link between CLEC3A and this signalling axis. Western blot analysis confirmed that parental cell lines (MCF7 and ZR‐75.1) exhibited markedly lower phosphorylation levels of AKT and its downstream target GSK3β compared to endocrine‐resistant cell lines (MCF7‐LHD and ZR‐75.1‐LHD) (Figure 7M). To directly assess whether CLEC3A regulates this critical signalling network, we employed siRNA‐mediated knockdown of CLEC3A in both resistant cell lines. Following CLEC3A suppression, we observed profound inhibition of AKT and GSK3β phosphorylation in resistant cells (Figure 7N). To further investigate whether CLEC3A‐mediated endocrine therapy resistance relies on the PI3K‐AKT signalling pathway, we employed the AKT inhibitor MK2206 to suppress PI3K‐AKT activity in endocrine‐resistant BC cells. In both MCF7‐LHD and ZR75.1‐LHD cell lines, CLEC3A knockdown failed to further inhibit cell proliferation following PI3K‐AKT pathway suppression (Figure 7O,P). These results suggest that CLEC3A promotes endocrine therapy resistance through activation of the PI3K‐AKT signalling axis. Collectively, these data revealed that the genes in ERS play important roles in promoting tumour malignant progression.

## 4 Discussion

Nearly 50% of ER+HER2− BC patients ultimately develop resistance to endocrine therapy. Endocrine resistance is a primary cause of treatment failure and mortality in ER+HER2− patients, thereby exacerbating the public health burden. Consequently, it is crucial to identify high‐risk endocrine resistance in ER+HER2− BC patients prior to the initiation of endocrine therapy to facilitate precision treatment. Although there have been numerous reports on the mechanisms underlying endocrine resistance [37, 38, 39], there is a lack of molecular models to predict the occurrence of resistance.

This study identified three genes that are upregulated in endocrine‐resistant cell lines and closely associated with survival through the comparative analysis of gene expression profiles between endocrine‐resistant and parental cell lines. We also developed an ERS risk stratification model based on these three key genes, effectively predicting the sensitivity of ER+HER2− BC patients to endocrine therapy. Furthermore, in vitro experiments revealed that the endocrine resistance‐related genes CLEC3A, PCDH10, and ST3GAL1 can influence the proliferation and migration capabilities of ER+HER2− BC cells. Mechanistically, CLEC3A functions as an upstream modulator of the PI3K‐AKT pathway, driving both the intrinsic survival advantages and drug‐tolerant phenotypes observed in endocrine‐resistant BC.

CLEC3A is a C‐type lectin protein that enhances tissue plasminogen activator, participates in cell adhesion, and plays a key role in the invasion and metastasis process [40, 41]. High expression of CLEC3A in BC tumour tissues has been reported to be associated with lymph node metastasis and poor prognosis [42]. But the role of CLEC3A in endocrine resistance remains unclear. Here we found that CLEC3A was significantly upregulated in endocrine‐resistant BC and promotes cell proliferation and migration by activating the PI3K‐AKT pathway, which was frequently upregulated across multiple cancer types and represents a targetable route for therapeutic intervention [37, 43, 44]. Our study reveals that CLEC3A serves not only as a driver of endocrine therapy resistance but also as a predictive biomarker to identify suitable candidates for AKT inhibitor, a drug already approved for the treatment of endocrine‐resistant BC in clinical practice.

PCDH10, a member of the protocadherin family, exhibits context‐dependent functions in tumour biology. While initial studies suggested its role as a tumour suppressor by inhibiting cellular proliferation [45], conflicting findings have been reported: some investigations indicate that PCDH10 promotes tumour progression via enhanced migration and metastasis, without direct effects on proliferation [46]. However, our study reveals a previously uncharacterized oncogenic role of PCDH10 in endocrine‐resistant BC. We demonstrate that PCDH10 is significantly upregulated in endocrine‐resistant tumour models and actively drives resistance phenotypes, enabling proliferation under oestrogen‐deprived conditions—a hallmark of therapeutic stress. These findings expand the functional repertoire of PCDH10, identifying it as a pro‐oncogenic factor under specific tumour contexts. Moreover, our work highlights its potential as a therapeutic target for overcoming endocrine resistance.

ST3GAL1 is a sialyltransferase that catalyses the transfer of sialic acid from CMP‐sialic acid to substrates containing galactose, terminating the extension of glycan chains. Upregulated expression of ST3GAL1 in BC promotes tumorigenesis, and its overexpression facilitates CD55‐mediated immune evasion [47]. In ovarian cancer models, ST3GAL1 overexpression is associated with cell proliferation, migration, and paclitaxel resistance [48]. Furthermore, it has been linked to poor prognosis in gliomas [49], osteosarcomas [50], and BC [51, 52]. Here, we found that ST3GAL1 overexpression is also significantly associated with the cell migration of endocrine resistance BC, but the mechanism was unclear. It is reported that the GFRA1/RET signalling pathway is a key factor in resistance to endocrine therapy in ER+HER2− BC [53], and GFRA1 is a substrate for ST3GAL1‐mediated O‐linked sialylation [51]. So ST3GAL1 might promote sialylation of GFRA1, thereby activating the GFRA1/RET signalling pathway to enhance endocrine resistance in ER+HER2− BC. We will further explore this mechanism in subsequent studies.

To further understand the molecular mechanisms associated with endocrine resistance, we conducted a GSEA analysis on DEGs between resistant and parental cell lines. The analysis revealed that cell cycle‐related pathways were significantly upregulated in resistant patients, while anti‐tumour immune‐related pathways were significantly downregulated. Further analysis of the immune microenvironment indicated an increase in M2 macrophages and a reduction in CD8+ T lymphocytes and activated NK cells in endocrine‐resistant tumours, confirming that these tumours possess an immunosuppressive microenvironment. This suggests that tumours exhibiting endocrine resistance have characteristics of rapid proliferation coupled with immune evasion.

Currently, there is a lack of precise models for predicting endocrine therapy resistance in BC patients. Therefore, we developed a nomogram based on ERS‐related molecular features and clinical variables, significantly enhancing the predictive ability for sensitivity to endocrine therapy and prognosis in BC patients. Accurate prediction of endocrine therapy sensitivity is crucial for achieving personalised treatment. Additionally, since endocrine‐resistant patients often have an immunosuppressive microenvironment, the efficacy of monotherapy with immunotherapy may be limited, necessitating the exploration of new combination strategies to convert “cold” tumours into “hot” tumours, thereby improving the effectiveness of immunotherapy.

However, this study has several limitations. First, the identification of prognostic endocrine therapy resistant genes was based on the TCGA database and METABRIC database, which, due to the heterogeneity of tumours, may not represent all types of cancer. Second, we conducted in vitro experiments on endocrine therapy resistance genes (CLEC3A, PCDH10, and ST3GAL1), but these findings have not been validated in vivo or in human studies. Moreover, the mechanisms by which the genes CLEC3A, PCDH10, and ST3GAL1 contribute to the malignant progression of BC also require further exploration. We also plan to conduct follow‐up studies to address the limitations of this research and hope that, in the future, this model can be validated in ER+HER2− BC patients to assess its efficacy and provide a reference for predicting endocrine treatment failure in ER+HER2− BC patients. More importantly, with the development of drug delivery and targeting technologies [54, 55, 56], we will further explore drugs targeting CLEC3A, PCDH10, or ST3GAL1 to overcome endocrine resistance.

## Author Contributions

All authors participated in this research, including conception and design (K.Z., H.T., X.S., L.P.), data acquisition (L.P., L.Z., N.C.), data analysis and interpretation (K.Z., L.P., L.Z., N.C.), material support (H.T., X.L., J.Z.), study supervision (K.Z., L.P., H.T., X.S.), as well as drafting the article or critically revising (L.P., K.Z., H.T., X.S.). The final version was ensured and approved by all authors.

## Ethics Statement

This study was approved by the medical ethics committee of Sun Yat‐sen University Cancer Center (G2023‐161‐01).

## Conflicts of Interest

The authors declare no conflicts of interest.

## Supporting information


**Data S1.** Figures.

**Table S1.** Genes upregulated in MCF7‐LHD or ZR75.1‐LHD.

**Table S2.** Genes associated with DFS or OS.

## Data Availability

The data that support the findings of this study are openly available in TCGA database at https://cancergenome.nih.gov/.
