# Supplementary material for: Endocrine Resistance Score Based on Three Key Genes Predicts Prognosis and Reveals Potential Therapeutic Targets for ER+HER2− Breast Cancer
Source: Cell Prolif. 2025 Jul 15;59(3):e70100. doi: 10.1111/cpr.70100 (PMC12961559; doi:10.1111/cpr.70100)
Supplement: Supplementary file 1 — Data S1. Figures. [file CPR-59-e70100-s002.docx]

**Supplemental Figures.**


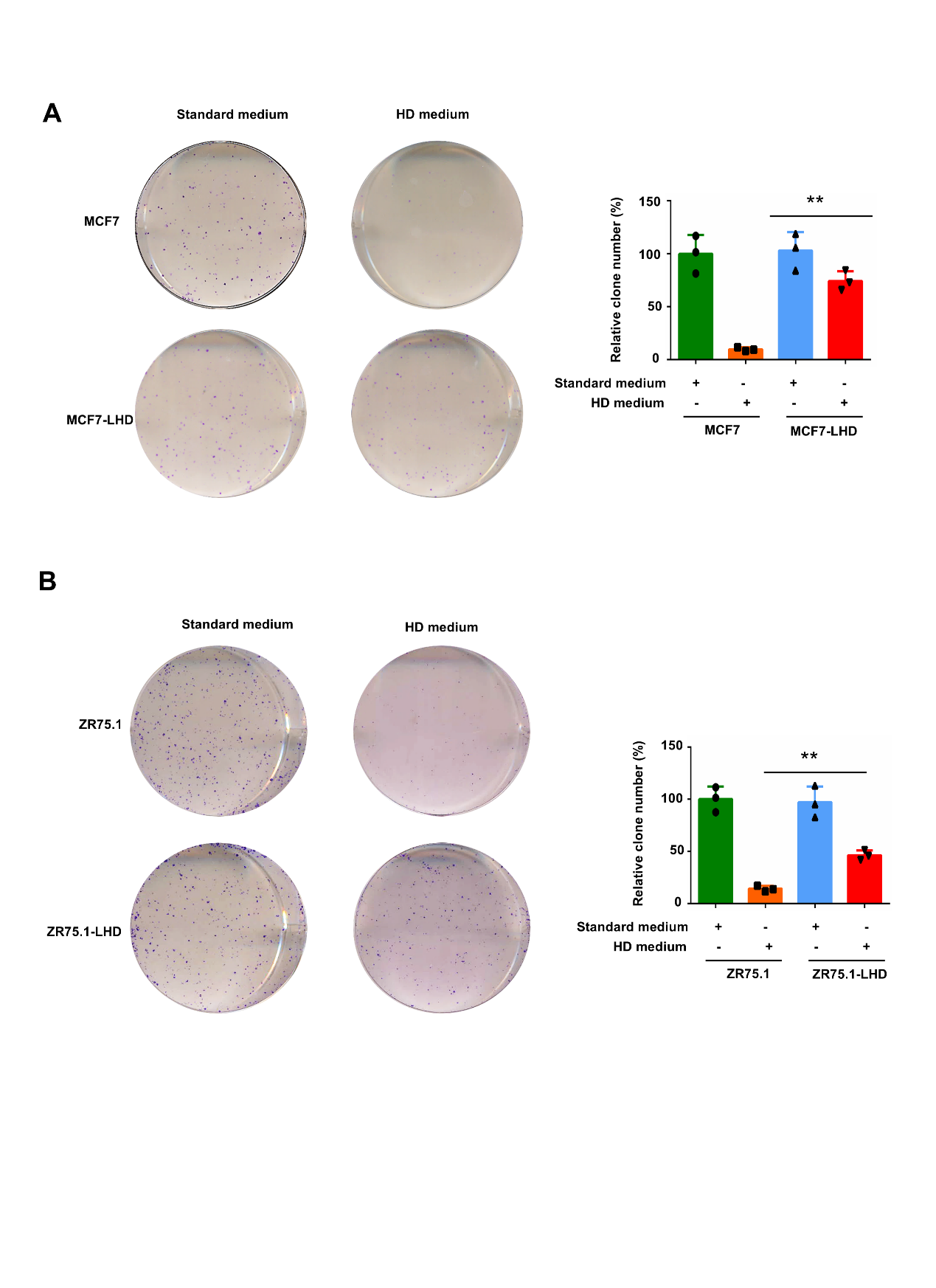


**Supplemental Figure 1. Validation of endocrine-resistant breast cancer cell Lines. A.** Colony formation assay of MCF7 and MCF7-LHD cells in standard and estrogen-deprived culture media. **B.** Colony formation assay of ZR75.1 and ZR75.1-LHD cells in standard and estrogen-deprived culture media. Error bars represent the standard error of the mean (*, p < 0.05; **, p < 0.01; ***, p < 0.001).

**
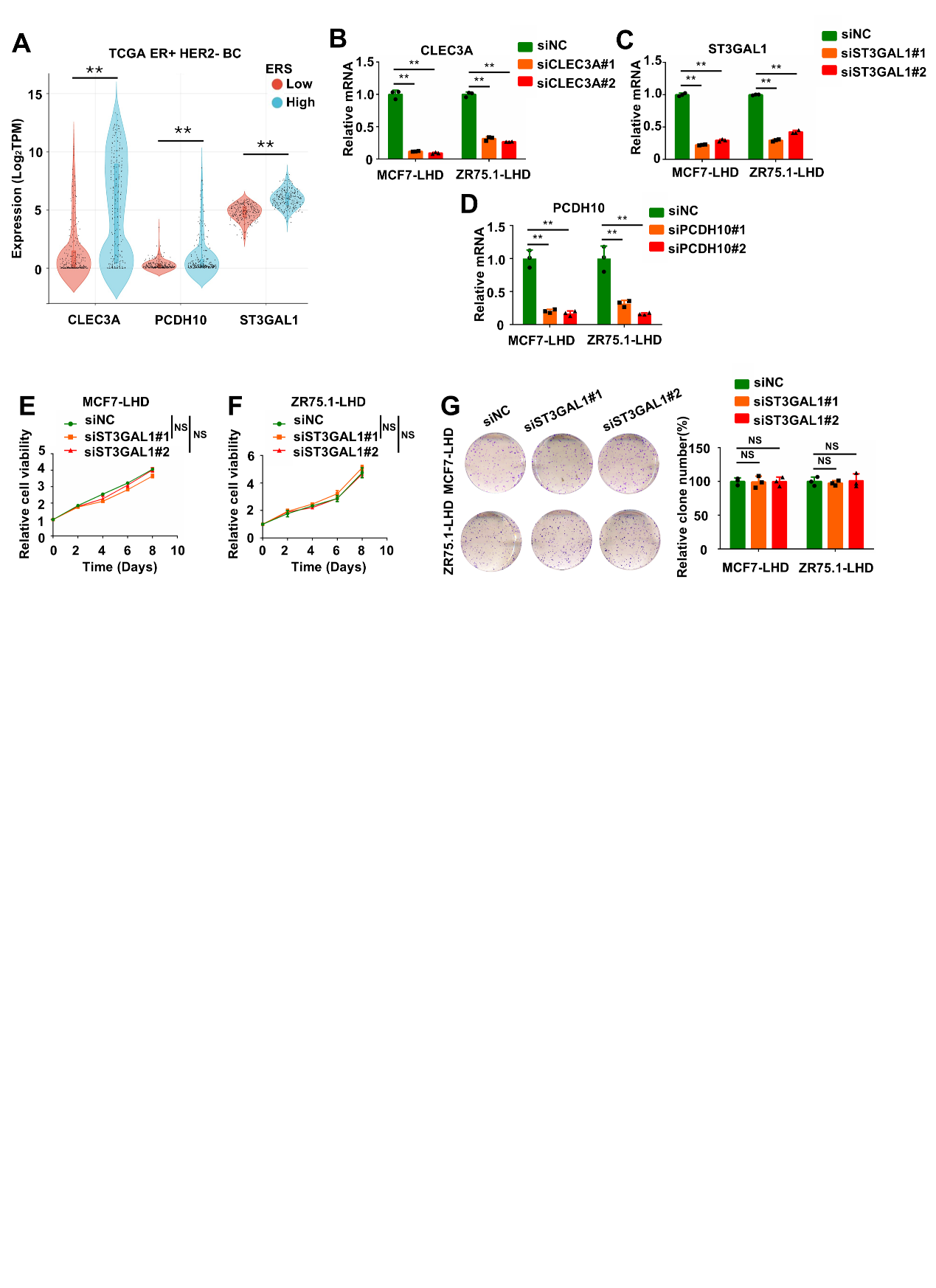
**

**Supplemental Figure 2. In vitro exploration of the functional roles of key genes associated with endocrine resistance. A.** Comparison of CLEC3A, PCDH10, and ST3GAL1 expression between ERS-high and ERS-low patients in the TCGA database. **B, C,** **D.** Validation of gene knockdown effects for CLEC3A, ST3GAL1 and PCDH10 in endocrine-resistant cell lines. **E, F.** Knockdown of ST3GAL1 in MCF7-LHD and ZR75.1-LHD cell lines results in no significant alteration of cell proliferation. **G.** Knockdown of ST3GAL1 in MCF7-LHD and ZR75.1-LHD cell lines results in no significant alteration of colony formation. Error bars represent the standard error of the mean (*, p < 0.05; **, p < 0.01; ***, p < 0.001).
